# Synthesis and application of a phenazine class substrate for high-throughput screening of laccase activity

**DOI:** 10.1007/s00253-023-12958-7

**Published:** 2024-01-09

**Authors:** Justinas Babinskas, Jerica Sabotič, Inga Matijošytė

**Affiliations:** 1 Life Sciences Center, Institute of Biotechnology, Sector of Applied Biocatalysis, Vilnius University, Saulėtekio ave. 7, Vilnius, LT-10257 Lithuania; 2 Department of Biotechnology, Jožef Stefan Institute, Jamova cesta 39, Ljubljana, 1000 Slovenia

**Keywords:** Biocatalysis, Laccase, High-throughput, Screening

## Abstract

Biocatalysis is one of the greatest tools for implementing the 12 principles of Green chemistry. Biocatalysts are bio-based, highly efficient and selective, operate at moderate conditions, and can be reused multiple times. However, the wider application of biocatalysts is plagued by a plethora of drawbacks, such as poor stability at operating conditions, inadequate efficiency of catalytic systems, a small number of commercially available biocatalysts, and a lack of substrates or methods for their discovery and development. In this work, we address the lack of suitable substrates for high-throughput screening of laccase by synthesising and investigating a newly developed phenazine-type substrate – Ferbamine. Investigation of Ferbamine pH and thermal stability indicated that its long-term stability in an aqueous medium is superior to that of commercially available substrates and does not require organic solvents. Ferbamine displayed convincing performance in detecting laccase activity on Ferbamine-agar plates in commercial laccase products and the collection of extracts from wild terrestrial fungi (42 species, 65 extracts), of which 26 species have not been described to have laccase activity prior to this work. Incubation of microorganisms on Ferbamine-agar plates showed its compatibility with live colonies. Ferbamine proved to be an easy-to-use substrate, which could be a great addition to the toolbox of methods for the functional analysis of laccases.

**Supplementary Information:**

The online version contains supplementary material available at 10.1007/s00253-023-12958-7.

## Keypoints

*A new phenazine-type substrate (Ferbamine) was developed for laccase activity assays.*

*Its pH and thermal stability in phosphate buffer exceeds that of ABTS.*

*Ferbamine screening method was validated using commercial laccase and environmental samples.*

**Supplementary Information:**

The online version contains supplementary material available at 10.1007/s00253-023-12958-7.

## Introduction

So far, high-throughput screening methods are the most powerful tools for discovering enzymes (Longwell et al. 2017). These methods are highly dependent on two factors: the origin of the enzyme and the character of the enzyme’s functional analysis. Sources of enzymes can be protein extracts from wild-type microorganisms, recombinant proteins, metagenomic libraries, engineered enzymes, etc. (Jacques et al. 2017). Enzyme functional analysis may be performed by the qualitative methods carried out on agar-substrate Petri plates or 96-well plates, zymograms or in silico; or by the quantitative methods such as spectroscopic, electrochemical or calorimetric. Both types of functional analysis require substrates for purposive enzymes (Reymond 2012). Unfortunately, many enzymes with high industrial potential lack proper substrates to follow their activities in processes and need high selectivity, sensitivity, high water-solubility and wide range if operational conditions of these substrates (Bisswanger 2014). Laccases are one of these prominent enzymes with a limited spectrum of substrates for functional analysis methods.

**Fig. 1:**
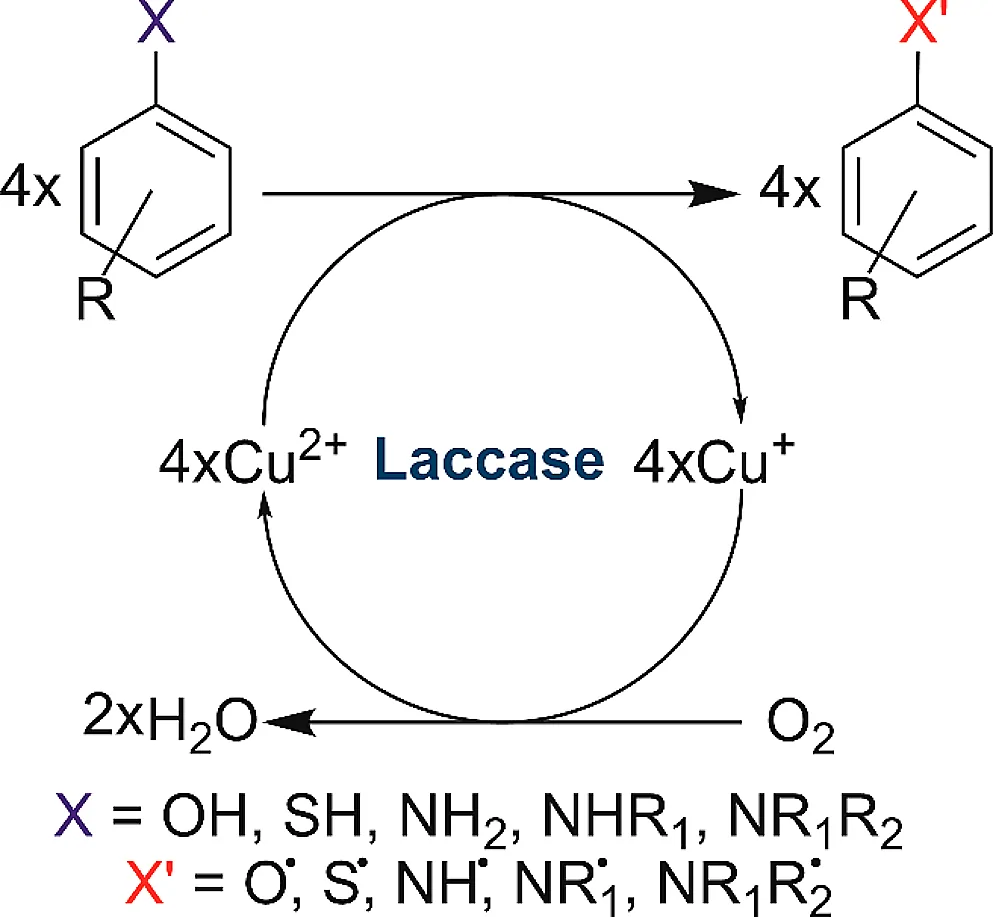
Simplified laccase catalysis reaction

Laccases are glycosylated multi-copper oxidases (EC 1.10.3.2) that belong to the small family of multi-copper oxidases (MCOs). Their active site contains four Cu ions, which are categorised according to their spectroscopic properties into Type 1 (T1), Type 2 (T2) and binuclear Type 3 (T3) Cu centres. Their natural substrates are aromatic compounds containing at least one functional hydroxyl, thiol, primary, secondary or tertiary amine group (Fig. 1) (Jones and Solomon 2015).

Furthermore, employing so-called mediators, low molecular weight electron transfer agents, makes a wide range of non-phenolic derivatives suitable substrates for laccases (Call and Mücke 1997). In general, the substrates of laccases can be classified into four classes according to the functional group coupled to the aromatic ring: hydroxyls (-OH), amines (-NH_2_, -NHR, -NRR’), thiols (-SH) and hybrids, i.e., substrates containing a combination of functional groups (Janusz et al. 2020). Hydroxyl substrates are commonly used for laccase functional analysis due to their water solubility, commercial availability, ease of use and low price. However, hydroxyl substrates have two significant disadvantages: limited structural variety and high oxidation potential. Structure variety is limited because laccases cannot oxidise aromatic ethers (-OR). The second disadvantage of the hydroxyl substrate is related to its electron-donating property. In general, the higher the electron density in the conjugated system, the more susceptible the conjugated system is to oxidation. Substrates with a single hydroxyl group have an average oxidation potential of 600 mV (Xu 1996), while the oxidation potential of laccases varies between 410 mV and 810 mV (Singh et al. 2015); therefore, they are not suitable for all laccases. To increase the electron density in the aromatic system, other functional groups with low steric effect, such as methoxy (2-methoxy phenol, 2,6-dimethoxy phenol, etc.) or other groups (1,2-benzenediol, 1,4-benzenediol, 1,2,3-benzene-triol) might be used. Still, their electronic effect must be electron-donating or neutral and be in para or ortho positions to the -OH group. The impact of electron-withdrawing groups must be compensated with other electron-donating groups (Pardo and Camarero 2015). Thiol substrates are not recommended to use due to their volatility and toxicity. Additionally, the thiol group has weak electron-donating properties and is prone to complexation with metal ions, light-triggered radical cleavage, oxidation, and disulphide bridge formation (Schlippert et al. 2016). Substrates containing an amine group can have a wide structural variety because the amine group has three suitable forms – primary, secondary, and tertiary. Amines have better electron-donor properties than hydroxyls (Sousa et al. 2014). The excess electronic density in the conjugated system also promotes faster autoxidation of the substrate. Fortunately, the rate of autoxidation can be limited by using *N*-substituted amines. These substituting groups must be electron-donating and have a low steric effect or weak electron-withdrawing groups; for example, methyl, ethyl, ethanol, etc. Tertiary amines can have two different substituted groups (Chung and Su 2009). Also, amine groups have a unique stabilisation effect – they can form highly stable organic salts.

Currently, the compounds most commonly used for functional analysis of laccases are the hybrid substrates: 2,2’-azino-bis(3-ethylbenzothiazoline-6-sulphonic acid) under the trivial name ABTS and 4-hydroxy-3,5-dimethoxybenzaldehyde azine known as syringaldazine (Call and Mücke 1997; Lucas et al. 2017). In short duration measurements, their performance is well studied, and the stability of the compounds is not an issue. But, it is known that ABTS solution stability is highly sensitive to temperature, pH, and even irradiation (Ozgen et al. 2006; Konan et al. 2016). Function-based screening methods usually need multiple-day microorganism cultivation (wild types, recombinant gene expression in hosts, genomic or metagenomic libraries, etc.) at elevated temperatures or varying pH, which could result in false positives. Therefore, ABTS plate’s incubation is performed only at room temperature (Amutha and Abhijit 2015; Senthivelan et al. 2019). Syringaldazine has higher stability than ABTS, but it is water-insoluble, thus, requires organics solvents for its use (Farnet et al. 2010). The substrates applied for laccase functional analysis need more stability for long-term high-throughput screening methods. Therefore, based on the properties of the functional groups of potential laccase substrates, we have determined that amine substrates should be the most suitable for the long-term and specifically featured high-throughput methods. In this work, we present the synthesis and investigation of the novel substrate with the given trivial name Ferbamine.

## Materials and methods

### Materials

*N*,*N*-Dimethyl-*p*-phenylenediamine dihydrochloride, 98% (Fisher Scientific chemicals, Fairlawn, USA); (NH_4_)_2_S_2_O_8_ – ammonium persulfate, reagent grade, 98% (Merck, Rahway, USA); Na_2_CO_3_ – sodium carbonate, ACS reagent, anhydrous, > 99.5%, powder (Merck, Rahway, USA); ethyl acetate, 99+% extra pure (Acros Organics, Geel, Belgium); agar, bacteriological grade (Acros Organics, Geel, Belgium); GMO-free soya peptone (Oxoid, Basingstoke, UK); methanol, >=99.9%, analytical reagent grade (Merck, Rahway, USA); sodium acetate trihydrate (Roth, Karlsruhe, Germany); Luria-Bertani Broth, Miller (Fisher Scientific, Schwerte, Germany); laccase – Novozymes^®^ 51,003 (Novozymes, Bagsværd, Denmark); glycerol 85% (Fluka, Buchs, Switzerland); yeast extract granulated (Merck, Rahway, USA); NaClO – sodium hypochlorite, 5% (Acros Organics, Geel, Belgium); NaBH_4_ – sodium borohydride (Fluka, Buchs, Switzerland); hydrogen peroxide 30% G.R. (Lach:ner, Neratovice, Czechia); chloroform, environmental grade, 99.8+%, stabilised with ethanol (Alfa Aesar, Ward Hill, USA); toluene (Sigma-Aldrich, Darmstadt, Germany); dimethyl sulfoxide (Merck, Rahway, USA); *N*,*N*-dimethylformamide, 99.8%, Extra dry over molecular sieve, AcroSeal^®^ (Acros Organics, Geel, Belgium); Metol – 4-(methylamino)phenol sulfate (Merck, Rahway, USA); cyclohexane, HPLC grade (Acros Organics, Geel, Belgium); diethyl ether, 99+%, pure, stabilised with BHT – butylated hydroxytoluene (Acros Organics, Geel, Belgium); tetrahydrofuran, 99.9% extra pure, anhydrous, stabilised with BHT (Acros Organics, Geel, Belgium); trypticase soy broth (BD, Franklin Lakes, USA), nutrient broth (Oxoid, Basingstoke, UK), P_2_O_5_ – phosphorus pentoxide, 99+%, for analysis (Acros Organics, Geel, Belgium); Thin-layer chromatography (TLC) plates 0.2 mm aluminium oxide 150 F254 type T (Merck, Rahway, USA).

### The synthesis of the Ferbamine at optimal conditions

The solution consisting of *N*,*N*-dimethyl-*p*-phenylenediamine dihydrochloride – DMPD•2HCl (7.53 g, 36 mmol) and 100 ml dH_2_O was prepared in a 400 ml beaker with a thermometer and magnetic stir bar. While stirring, small portions of Na_2_CO_3_ (3.816 g, 36 mmol in total) were added to the solution to neutralise HCl. The solution was cooled down to 0–5 °C in an ice bath. (NH_4_)_2_S_2_O_8_ (8.2 g, 36 mmol) was dissolved in 40 ml dH_2_O, cooled to 0–5 °C in an ice bath, and added dropwise to the DMPD solution while maintaining temperatures below 5 °C. After 3 h of mixing, the reaction mixture was warmed to 20 °C. Black precipitate was collected using the Büchner filtration system and washed with 4 × 100 ml of dH_2_O. The precipitate was dried over P_2_O_5_ in an exicator for 24 h. 150 mg of dry precipitate and 150 ml of EtOAc were added to a 250 ml round-bottom flask with an Allihn condenser and magnetic stir bar. The slurry was refluxed for 1 h and cooled down to 20 °C. The dark slurry was collected using the Büchner filtration system, washed with 4 × 50 ml of EtOAc, and dried in the open air. 50 mg of dry precipitate and 100 ml of 5% MeOH/dH_2_O was added to a 250 ml round-bottomed flask with an Allihn condenser and magnetic stir bar. The mixture was refluxed for 4 h and cooled down to 20 °C. Insoluble residues were filtered using a Büchner filtration system, and Ferbamine was collected by evaporating the filtrate at 55 °C under reduced pressure. The yield was 0.92%. The melting point of Ferbamine (not purified) was assigned to be 160–164 °C when it decomposed. ^1^H NMR (d_6_-DMSO, 400 MHz): δ 8.88 (s, 1H), 8.14 (s, 1H), 8.05 (d, *J* 9.56 Hz, 1H) 7.93 (d, *J* 8.72 Hz, 1H), 7.69 (d, *J* 8.24 Hz, 2 H), 7.01 (d, *J* 8.76 Hz, 2 H), 6.87 (s, 1H), 3.23 (s, 6 H,), 3.06 (s, 6 H), 1.23 (s, 6 H). ^13^C NMR (d_6_-DMSO, 100 MHz): δ 152.03, 151.72, 149.05, 146.10, 140.90, 137.77, 134.23, 132.39, 130.40, 126.25, 125.93, 121.59, 113.04, 112.91, 109.58, 100.37, 34.79, 34.08, 29.46. HRMS *m/z*: calculated 397.2135, found 397.2132 [M]^+^ (C_24_H_25_N_6_^+^); calculated 411.2292, found 411.2289 [M]^+^ (C_25_H_27_N_6_^+^); calculated 383.1978, found 383.1979 [M + H]^+^ (C_24_H_25_N_6_ + H)^+^. Attenuated total reflectance Fourier-transform infrared spectroscopy measurements (ATR-FTIR): 3365, 3227, 3043, 2927, 2811, 1610, 1569, 1520, 1456, 1449, 1339, 1361, 1290, 1226, 1196, 1169, 1129, 1068, 946, 820, 755, 618, 548 cm^-1^. Thin-layer chromatography (TLC) analysis procedure covered the sample elution on a 0.2 mm aluminium oxide 150 F254 type T plate (Merck, Rahway, USA) using MeOH and EtOAc 1:1 (v/v) mixture as a mobile phase. Plates were placed in a desiccator with iodine to visualise the plates.

### Preparation of fungal extracts

The fruiting bodies of 42 different basidiomycete species were collected from forest stands or meadows at various locations in western and central Slovenia, identified to species level following taxonomic classification of the Index Fungorum database (Index Fungorum Partnership 2023) and stored at -20 °C. The frozen fruiting bodies were thawed and homogenised with a press to obtain a crude extract, to which sterile 20× phosphate-buffered saline (100 mM Na_2_HPO_4_, 18 mM KH_2_PO_4_, 1.37 M NaCl, 27 mM KCl, pH 7.4) was added to obtain a 1× phosphate-buffered extract. Centrifugation (10,000× g, 10 min, 4 °C) removed the insoluble material. After dialysis against phosphate-buffered saline (3.5 kDa cut-off), the crude aqueous extracts were concentrated 10-fold by ultrafiltration (3 kDa cut-off). Finally, the fungal extracts were filtered and sterilised using 0.2 μm filter. The dialysis and concentration steps were omitted for the extracts numbered 39 to 65.

### Preparation of Ferbamine-agar plates

*Ferbamine-agar plate (37.5 µg ml*^*-1*^, *20 ml) with wells*. Sterile 9% (w/w) agar stock solution was prepared by mixing 0.3 g of agar with 3.4 ml of dH_2_O and autoclaving it for 20 min at 121 °C temperature. The agar stock was warmed to 80–85 °C and mixed with 16.6 ml of 45 µg ml^-1^ Ferbamine solution. The mixture was stirred at 80–85 °C until it became homogenous. A polypropylene 200 µl PCR 96-well plate was placed in a Petri dish as a good template. Hot Ferbamine-agar solution was added into the Petri dish and let to cool down to ambient temperature. After the agar solidified, the 96-well plate was removed. Ferbamine-agar plate composition: 37.5 µg ml^-1^ Ferbamine, 1.5% agar.

*Ferbamine-LB agar plates for* Escherichia coli *and* Pseudomonas putida. Luria-Bertani (LB) Miller agar plates containing various Ferbamine amounts (0-37.5 µg ml^-1^) were prepared by mixing LB agar stock solution with different volumes of 45 µg ml^-1^ Ferbamine solution. LB agar stock was prepared by mixing 2.94 g of LB broth, 1.76 g of agar and 20 ml of dH_2_O, and autoclaving it at 121 °C temperature for 20 min. Volumes for mixing Ferbamine solution, LB agar stock and sterile dH_2_O (for dilution) are presented in Table 1. Ferbamine-LB agar plate compositions: 1% tryptone, 0.5% yeast extract, 1% NaCl, 1.5% agar, 4.5–37.5 µg ml^-1^ Ferbamine. The control plate was prepared omitting the Ferbamine.

**Table 1:** Volumes of stock solutions for 0-37.5 µg ml^-1^ Ferbamine-growth medium agar plate preparation

Ferbamine concentration [µg ml^-1^]	Ferbamine solution [ml]	Medium agar stock [ml]	dH_2_O [ml]
37.5	16.6	3.4	0
22.5	10	3.4	6.6
4.5	2	3.4	14.6
0 (control)	0	3.4	16.6

*Ferbamine-TS agar plates for* Bacillus pumilus *and* Rhodococcus erythropolis. Trypticase-soy (TS) agar plates containing various Ferbamine amounts (0-37.5 µg ml^-1^) were prepared by mixing TS agar stock solutions with different volumes of 45 µg ml^-1^ Ferbamine solution. TS agar stock was prepared by mixing 3.53 g of trypticase soy broth, 1.76 g of agar and 20 ml of dH_2_O and autoclaving it at 121 °C temperature for 20 min. Volumes for mixing Ferbamine solution, TS agar stock and sterile dH_2_O (for dilution) are presented in Table 1. Ferbamine-TS agar plate compositions: 3% trypticase soy broth, 1.5% agar, 4.5–37.5 µg ml^-1^ Ferbamine. The control plate was prepared omitting the Ferbamine.

*Ferbamine-YEPG agar plates for yeasts* Kluyveromyces marxianus, Kluyveromyces lactis, Pichia pastoris *and* Saccharomyces cerevisiae. Yeast extract-peptone-glycerol (YEPG) plates containing various Ferbamine amounts (0-37.5 µg ml^-1^) were prepared by mixing YEPG agar stock solutions with different volumes of 45 µg ml^-1^ Ferbamine solution. YEPG agar stock was prepared by mixing 2.35 g of peptone, 1.18 g of yeast extract, 2.76 g of 85% glycerol, 1.76 g of agar and 20 ml of dH_2_O, and autoclaving it at 121 °C temperature for 20 min. Volumes for mixing of Ferbamine solution, YEPG agar stock and sterile dH_2_O (for dilution) are presented in Table 1. Ferbamine-YEPG agar plate compositions: 2% peptone, 1% yeast extract, 2% glycerol, 1.5% agar, 4.5–37.5 µg ml^-1^ Ferbamine. The control plate was prepared omitting the Ferbamine.

*Ferbamine-nutrient agar plates for bacteria* Achromobacter denitrificans, Delftia acidovorans, Pseudomonas aeruginosa, Alcaligenes faecalis *and* Acinetobacter calcoaceticus. Nutrient agar plates containing various Ferbamine amounts (0-37.5 µg ml^-1^) were prepared by mixing nutrient agar stock solutions with different volumes of 45 µg ml^-1^ Ferbamine solution. Nutrient agar stock was prepared by mixing 1.53 g of nutrient broth, 1.76 g of agar and 20 ml of dH_2_O and autoclaving it at 121 °C temperature for 20 min. Volumes for mixing Ferbamine solution, nutrient agar stock and sterile dH_2_O (for dilution) are presented in Table 1. Ferbamine-nutrient agar plate compositions: 0.1% lab-lemco, 0.5% peptone, 0.2% yeast extract, 0.5% NaCl, 1.5% agar, 4.5–37.5 µg ml^-1^ Ferbamine. The control plate was prepared omitting the Ferbamine.

### Ultraviolet-visible (UV-VIS) light spectroscopy of native, partially reduced or oxidized Ferbamine solutions

Ferbamine stock solution was diluted from 45 µg ml^-1^ to 7.5 µg ml^-1^ with dH_2_O, 1% NaClO or 0.1 g ml^-1^ NaBH_4_, and incubated at 20 °C for 30 min. Spectra measurements were carried out in a quartz cuvette with light path of 1.000 cm, at 20 °C temperature, using a spectrophotometer Spekol 2000 (Analytik Jena, Jena, Germany). The measurement range was 320–750 nm with 2 nm increments. Reference measurement was performed with dH_2_O.

### Thermal and pH stability of Ferbamine

The analysis was performed using UV-VIS spectroscopy. Spectra measurements were carried out in a glass cuvette with light-path of 1.000 cm, at 20 °C temperature, using a spectrophotometer Spekol 2000 (Analytik Jena, Jena, Germany). The measurement range was 380–750 nm with 2 nm increments. 1 ml of 45 µg ml^-1^ Ferbamine solution was diluted with 5 ml of 60 mM potassium phosphate buffer of pH 3.5, 4.0, 5.0, 6.0, 7.0, 8.0; or dH_2_O to set the control solution. The initial UV-VIS spectra were measured, and initial absorption maximums (*λ*_*Max*_) were determined for each solution. Solutions were divided into three parts (3 × 2 ml) and incubated at three temperatures (20, 30 and 37 °C) for 5 weeks. Consecutive UV-VIS spectra measurements were performed every week. Stability was calculated (1) and defined as the percentage of remaining absorption at the *λ*_*Max*_.

1 $$Stability \left(\%\right)=\left(1-\frac{{A}_{0}-{A}_{t}}{{A}_{0}}\right)*100 \%$$

Where *A*_*0*_ – initial absorption at *λ*_*Max*_, *A*_*t*_ – absorption after *t* number of weeks, at the initial *λ*_*Max*_.

### Functional analysis of Novozymes^®^ 51,003 laccase on Ferbamine-agar plate

Sterile 9% (w/w) agar stock solution was prepared by mixing 0.3 g of agar with 3.4 ml of dH_2_O and autoclaving it for 20 min at 121 °C temperature. The agar stock was warmed to 80–85 °C and mixed with 2 ml of 45 µg ml^-1^ Ferbamine solution and 14.6 ml of dH_2_O. The mixture was stirred at 80–85 °C until it became homogenous. Hot Ferbamine-agar solution was added into the Petri dish and let to cool down to ambient temperature. The commercially available laccase Novozymes^®^ 51,003 was added (1–4 µl) onto the Ferbamine plate and incubated for 20 h, at 20 °C. Ferbamine-agar plate composition: 4.5 µg ml^-1^ Ferbamine, 1.5% agar.

### The analysis of Ferbamine effect on microorganisms

Fresh colonies of *E. coli* DH5-α, *P. putida* TBSLT100 were transferred onto (0-37.5 µg ml^-1^) Ferbamine-LB agar plates and incubated at 37 °C overnight for 10 h. Fresh colonies of *B. pumilus* and *R. erythropolis* DSMZ 312 (DSMZ public collection, Braunschweig, Germany) were transferred onto (0-37.5 µg ml^-1^) Ferbamine-TS agar plates and incubated at 30 °C for 168 h (7 days). Fresh colonies of *K. marxianus* BKM Y-719, *K. lactis* MD2/1, *K. phaffii* (*Pichia pastoris*) GS115 and *S. cerevisiae* AH22-214 were transferred onto (0-37.5 µg ml^-1^) Ferbamine-YEPG agar plates and incubated at 30 °C up to 168 h (7 days). Fresh colonies of *D. acidovorans*, *A. faecalis* and *A. calcoaceticus* BT8 were transferred onto 0 µg ml^-1^ and 37.5 µg ml^-1^ Ferbamine-nutrient agar plates, and incubated at 30 °C for 48 h. Fresh colonies of *A. denitrificans* and *P. aeruginosa* A2 were transferred onto 0 µg ml^-1^ and 37.5 µg ml^-1^ Ferbamine-nutrient agar plates, and incubated at 37 °C for 48 h.

### UV-VIS spectroscopic laccase activity assay with Metol

Laccase activity was measured by observing the formation of the oxidised Metol (*N*-methyl-*p*-aminophenol sulfate) product at 542 nm. Measurements were performed in a glass cuvette, using UV-VIS spectrophotometer Spekol 2000 (Analytik Jena, Jena, Germany). Measurement durations were 60 s, with 2 s increments, and the start was delayed by 5 s for mixing. The substrate solution contained 2.0 ml 50 mM of Metol in dH_2_O and 0.5 ml of 50 mM pH 5.0 sodium acetate buffer. The reaction was initiated by adding 0.5 ml of enzyme solution to the substrate. Reference measurement was performed by adding 0.5 ml of 50 mM sodium acetate buffer (pH 5.0) to the substrate solution. Laccase activity [U] was calculated using (2). 1 U ml^-1^ was defined as 1 µmol of Metol oxidised per 1 min at pH 5.0 and 20 °C.

2 $$U=\frac{{tg}\alpha }{\epsilon \mathrm{*}l}\mathrm{*}\frac{{V}_{R}}{{V}_{E}}\mathrm{*}60\mathrm{*}{D}_{E}$$

Where *U* – laccase activity [U ml^-1^ or µmol ml^-1^ min^-1^], *tg α* – absorption slope [Abs s^-1^], *ε* – molar extinction coefficient of Metol oxidation product at 542 nm [2 mM^-1^ cm^-1^], *l* – length of light path [cm], *V*_*R*_ – reaction volume in the cuvette [3 ml], *V*_*E*_ – enzyme solution volume in the reaction mixture [0.5 ml], 60 – seconds to minutes conversion factor [s min^-1^], *D*_*E*_ – enzyme dilution before the measurement.

### Attenuated total reflectance Fourier-transform infrared spectroscopy (ATR-FTIR) analysis of Ferbamine synthesis product

Spectrophotometer ALPHA I (Bruker, Billerica, USA) was equipped with Platinum-ATR single reflectance diamond module. 64 accumulative scans with 2 cm^-1^ resolutions were conducted from 400 to 4000 cm^-1^ for background and sample measurements. Sample material dissolved in MeOH was analysed as a thin film on the ATR crystal: 3 µl of a sample solution was repeatedly applied and evaporated on the ATR crystal at ambient temperature. This process was repeated until real-time single scan spectra stabilized. After that, full spectra measurement was taken. OPUS 7.2 (Bruker, Billerica, USA) and SpectraGryph v1.2.16.145 (Menges 2016) software was used to analyse the data.

## Results

### Development of the Ferbamine synthesis

Oxidizing agent ((NH_4_)_2_S_2_O_8_) to DMPD ratio was varied from 0.90 to 1.1 by the step increase of 0.05 to achieve the best yield. It was established that a 1.0 equivalent is an optimum amount because the usage of 1.05 and higher equivalents led to the increased formation of by-products. Hydrogen peroxide was also tested as an oxidizing agent, but it formed many new derivatives as by-products.

In order to remove by-products formed during oxidation 50 mg of the precipitate was refluxed with 10 ml of a particular organic solvent: ethyl acetate (EtOAc), diethyl ether, toluene, cyclohexane, tetrahydrofuran, or chloroform. TLC analysis indicated that EtOAc was the most effective solvent to dissociate the precipitate from the by-products. Flash and gravity silica gel chromatography were employed for the supreme removal of unwanted compounds. Most of the compounds exhibited intense tailing even at low concentrations preventing the removal of by-products.

The second step of the overall synthesis process was carried out by refluxing 50 mg of the dry precipitate obtained in the first part of synthesis with 100 ml of dH_2_O for 1–5 h at 100 °C. The obtained data revealed that the highest yield was attained after 4 h. A strategy to further increase the synthesis yield was explored by using organic solvents entirely or their mixtures with dH_2_O. Methanol (MeOH), ethanol, *N*,*N*-dimethylformamide and dimethyl sulfoxide were selected as organic solvents. Their volume in mixtures with dH_2_O was 1%, 3%, 5%, 10%, 50% and 90% (v/v). The reactions were carried out for 4 h and at 100 °C. When the solvent’s boiling point was lower than 100 °C, the reaction was carried out at its boiling point temperature. The TLC analysis results indicated that Ferbamine did not form in solvents with water content lower than 95%. The best overall yield (0.92%) was achieved using 5% MeOH.

### Thermal and pH stability of Ferbamine

**Fig. 2:**
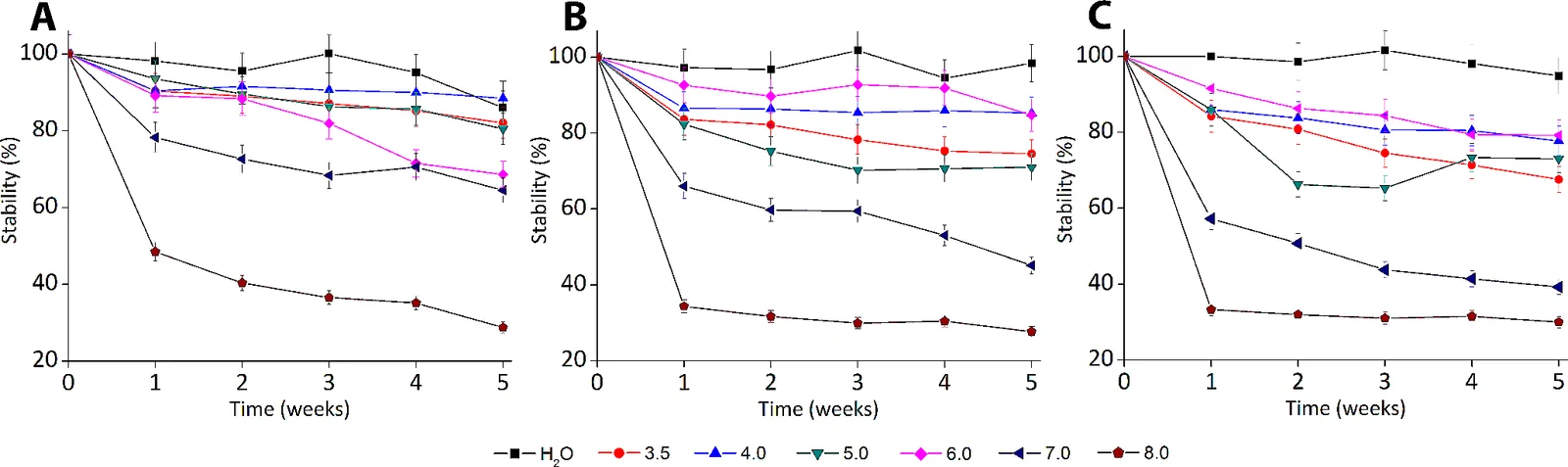
Thermal and pH stability of Ferbamine solution. Incubation temperatures: **A** 20 °C, **B** 30 °C, **C** 37 °C

In this set of experiments, 7.5 µg ml^-1^ Ferbamine was incubated in 50 mM Na-phosphate buffer solution in a range of pH 3.5-8.0 at 20, 30, or 37 °C. A solution of Ferbamine in dH_2_O (pH 5.7) was used as a reference to pursue the influence of buffer ions. UV-VIS spectra of the solutions were measured once a week. The stability of Ferbamine was calculated as the percentage of the remaining absorption relative to the starting absorption. The stability data is presented in Fig. 2.

According to the findings, the Ferbamine after 5 weeks at pH 3.5-6.0 retained from 67.6 to 85.2% of its original absorption. At pH 7.0, the absorption remained above 60% at 20 °C (64.5%); at 30 and 37 °C, it decreased to 45.1% and 39.3%, respectively. At pH 8.0 the absorption dropped below 50% after the first week of incubation, and it was 28–30% after five weeks. The absorbance of Ferbamine solution prepared in dH_2_O did not diminish below 86% at all temperatures.

### Application of Ferbamine for laccase activity screenings

**Fig. 3:**
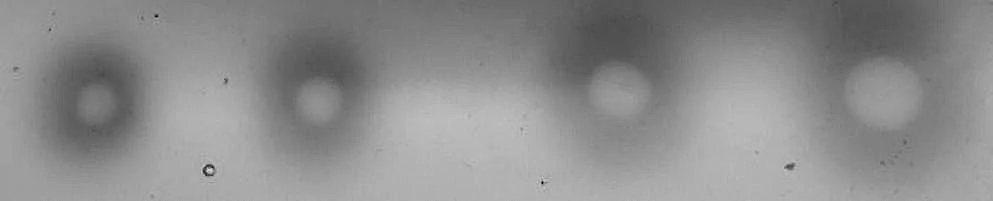
Functional analysis of Novozymes^®^ 51,003 laccase on the agar plates containing 4.5 µg ml^-1^ Ferbamine. Amount of the enzyme added, from left to right: 1, 2, 3, 4 µl. Incubation conditions: 20 h at 20 °C

We started with the commercially available laccase Novozymes^®^ 51,003. The spectrophotometric Metol assay measured its laccase activity to be 2.7 U ml^-1^. 1 U was defined as 1 µmol of Metol catalysed per 1 min at pH 5.0 and 20 °C. 1–4 µl of this enzyme was added to the plate containing Ferbamine at a concentration of 4.5 µg ml^-1^ and incubated at 20 °C for 20 h (Fig. 3).

The results indicated that the oxidation of Ferbamine catalysed by laccase produced a pale yellow area with a purple halo.

Further screening with Ferbamine as a substrate was applied to survey laccase activity in extracts from wild terrestrial fungi. The experiment was performed on 37.5 µg ml^-1^ Ferbamine-agar plates with wells: 2 µl of the extracts were added and incubated at 20 °C for 20 h. The obtained results of laccase activity were classified into three levels depending on the relative intensity of colour change: +++ significant; ++ moderate; + detectable. In parallel, spectrophotometric Metol assay also screened laccase activity in the extract collection. The results of both screening approaches and previous reports on the presence of laccase in the tested species are presented in Table 2.

**Table 2:** The results of laccase activity screening in the collection of extracts from terrestrial fungi

Extract No.^*a*^	Species	Assays		Previous reports on laccase^*c*^
		Metol [U ml^-1^]^*b*^	Ferbamine	
3	*Clitocybe nebularis*	9.00	**+++**	Yes (Asav 2021)
39	*Psilocybe cubensis*	5.40	**+++**	Yes (Lenz et al. 2020)
6	*Cortinarius violaceus*	5.25	**+**	No (Chen et al. 2003)
2	*Macrolepiota procera*	4.80	**+++**	Yes (Luis et al. 2004)
50	*Gomphidius glutinosus*	3.90	**-**	Not tested
53	*Pleurotus eryngii*	2.70	**+++**	Yes (Guo et al. 2017)
1	*C. nebularis*	2.55	**+++**	Yes (Asav 2021)
67	*Rhodocybe gemina*	1.65	**+**	Not tested
15	*Tricholoma tigrinum*	1.65	**+**	Not tested
66	*Tricholoma saponaceum*	1.50	**++**	Yes (Khaund and Joshi 2014)
33	*Lentinula edodes*	1.13	**-**	Yes (Yano et al. 2009)
5	*Ganoderma lucidum*	1.05	**++**	Yes (Jeon et al. 2008)
55	*Gymnopilus penetrans*	0.90	**+**	No (Marr et al. 1986)
9	*Cortinarius caperatus* (*Rozites caperata*)	0.83	**+++**	Not tested
62	*Amanita vaginata*	0.83	**+**	Not tested
47	*Anthurus archeri* (*Clathrus archeri*)	0.75	**+**	Not tested
18	*Pleurotus ostreatus* (summer strain)	0.75	**+**	Yes (Park et al. 2014)
35	*Clitocybe geotropa*	0.68	**+**	Not tested
54	*Amanita spissa*	0.68	**-**	No (Chen et al. 2003)
70	*A. vaginata*	0.68	**-**	Not tested
51	*Tricholomopsis rutilans*	0.68	**-**	Not tested
59	*Clavariadelphus pistillaris*	0.60	**+**	Yes (Marr et al. 1986)
40	*Ramaria stricta*	0.53	**-**	Yes – ABTS, guaiacol; No – syringaldazine (Erden et al. 2009)
20	*P. ostreatus* (winter strain)	0.45	**+**	Yes (Park et al. 2014)
36	*Russula alutacea*	0.45	**+**	Not tested
57	*C. geotropa*	0.38	**-**	Not tested
31	*Paxilllus atrotomentosus*	0.38	**-**	Not tested
69	*Cortinarius multiformis*	0.30	**++**	Not tested
32	*Clitocybe gibba*	0.30	**-**	Not tested
28	*Lactarius necator*	0.23	**-**	Not tested
63	*Amanita rubescens*	0.15	**-**	No (Hutchison 1990)
58	*Craterellus cornucopioides*	0.15	**-**	Not tested
43	*P. ostreatus*	0.15	**-**	Yes (Park et al. 2014)
42	*Xerocomus badius*	0.15	**-**	No (Hutchison 1990)
65	*Agaricus bisporus*	0.08	**-**	Yes (Mayolo-Deloisa et al. 2011)
41	*A. rubescens*	0.08	**-**	No (Hutchison 1990)
42	*A. rubescens*	0.08	**-**	No (Hutchison 1990)
46	*P. atrotomentosus*	0.08	**-**	Not tested
24	*Amanita excelsa*	**-**	**+**	Not tested
16	*Macrolepiota rachodes*	**-**	**+**	No (Luis et al. 2004)

^*a*^ Extracts which did not show laccase activity in Metol and Ferbamine assays are excluded. They are included in Supplemental Table S1, together with photos of the Ferbamine plates.

^*b*^ 1 U is defined as 1 µmol of Metol catalysed per 1 min, at pH 5.0 and 20 °C

^*c*^ Yes – laccase gene or activity has been previously reported in the species; No – species has been tested, but laccase gene or activity was not detected; Not tested – species has not been tested for laccase genes or activity

Of the 65 protein extracts examined, 25 showed no laccase activity in the Metol and Ferbamine assays (excluded from Table 2; included in Supplemental Table S1). On the Ferbamine plates, six protein extracts showed significant laccase activity, three moderate and 13 detectable. Extract N°3 had the highest activity (9.00 U ml^-1^) and exposed the most significant colour intensity change. The lowest laccase activity detected by Ferbamine was 0.30 U ml^-1^ in extract N°69.

**Fig. 4:**
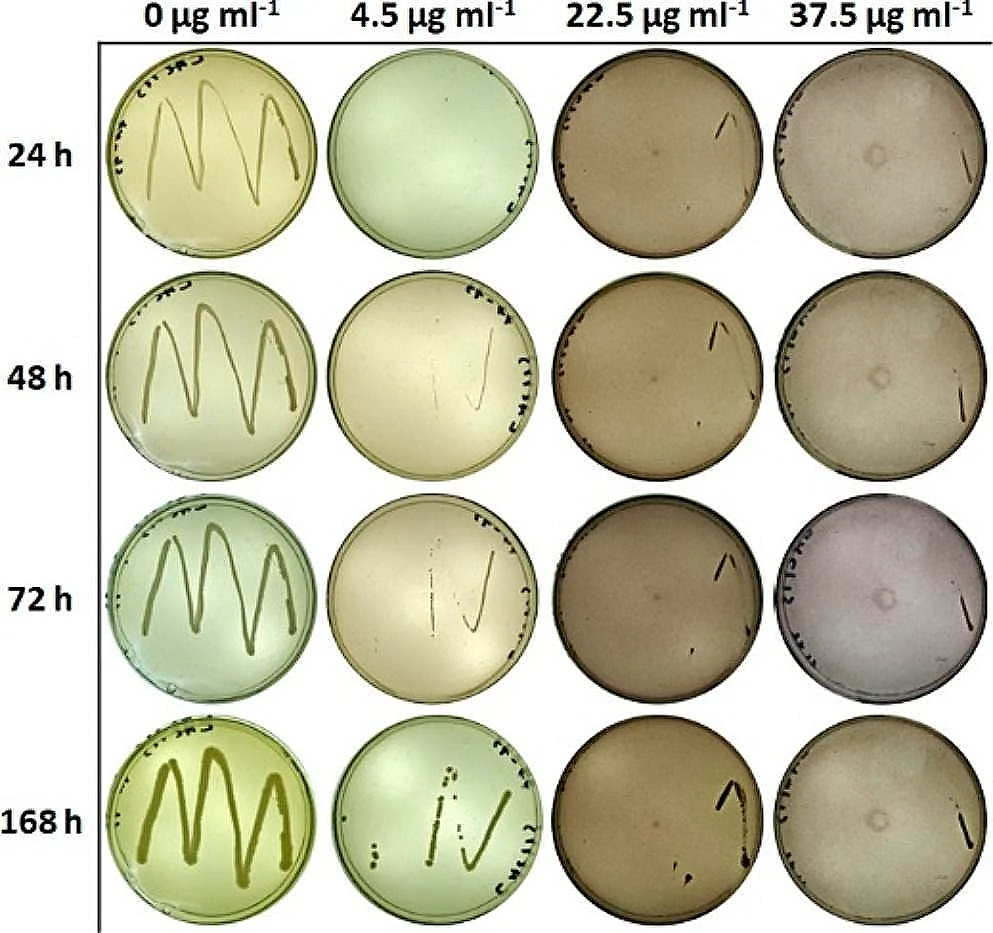
Results of Ferbamine concentration (0-37.5 µg ml^-1^) influence on the growth of *K. lactis*

The tests on the toxicity of Ferbamine to microorganisms were performed on two strains of Gram-negative bacteria (*E. coli*, *P. putida*), two Gram-positive bacteria (*R. erythropolis*, *B. pumilus*) and four strains of yeast (*K. marxianus*, *K. lactis*, *P. pastoris*, *S. cerevisiae*). Microorganisms were incubated for up to 168 h and at different temperatures: *E. coli*, *P. putida*, *B. pumilus* at 37 °C and other strains at 30 °C. Ferbamine concentrations in the plates were 4.5 µg ml^-1^, 22.5 µg ml^-1^, or 37.5 µg ml^-1^, and the plate omitting Ferbamine was used as a control. The tests revealed that Ferbamine did not affect the growth of *E. coli* and *P. putida* – colonies were fully grown after overnight incubation (10 h) on the plate with the highest Ferbamine concentration – 37.5 µg ml^-1^ (Supplemental Fig. S1). Data on the other strains (*B. pumilus*, *R. erythropolis*, *K. marxianus*, *K. lactis*, *S. cerevisiae* and *P. pastoris*) displayed a slight inhibition of growth by Ferbamine. After 168 h of incubation, little to no growth was observed on plates containing 22.5 µg ml^-1^ and 37.5 µg ml^-1^ of Ferbamine, while it was protracted on plates with 4.5 µg ml^-1^ concentration. The degree of inhibition was similar among all strains. The results for *K. lactis* are presented in Fig. 4, and the results for the other strains are presented in Supplemental Fig. S2.

The difference in inhibition between Gram-negative and Gram-positive microorganisms led to an assumption that Gram-negative bacteria were more resistant to Ferbamine. To test it, an experiment was carried out on five Gram-negative bacteria (*A. denitrificans*, *D. acidovorans*, *P. aeruginosa*, *A. faecalis*, *A. calcoaceticus*) from own collection (Vilnius University). Microorganisms were grown on 37.5 µg ml^-1^ Ferbamine-nutrient and control (omitting Ferbamine) plates at 37 °C for 48 h. The results revealed that microorganism growth on Ferbamine plates was only marginally slower than on control plates (Supplemental Fig. S3).

## Discussion

*N*,*N*-Dimethyl-*p*-phenylenediamine was chosen as the initial compound having two amine groups – the primary (-NH_2_) and tertiary (-*N*-(CH_3_)_2_) – and belongs to the class of phenylenediamine compounds. This class’s *ortho* and *para* variants exhibit similar properties to *p-* or *o-*hydroquinones. One of them is being easily oxidised under mild conditions. The product of phenylenediamine oxidation is benzoquinone diimine. The latter is readily reactive to participate in nucleophilic addition-type reactions with starting phenylenediamine or supplemented nucleophiles (Coulter et al. 2007; Sousa et al. 2014). These reactions are well known and have significant importance for producing dyes on an industrial scale (Berneth 2008). However, besides all the attempts (Sousa et al. 2014; Meyer and Fischer 2015), the oxidation of phenylenediamines and the following processes are not fully understood.

**Scheme 1:**
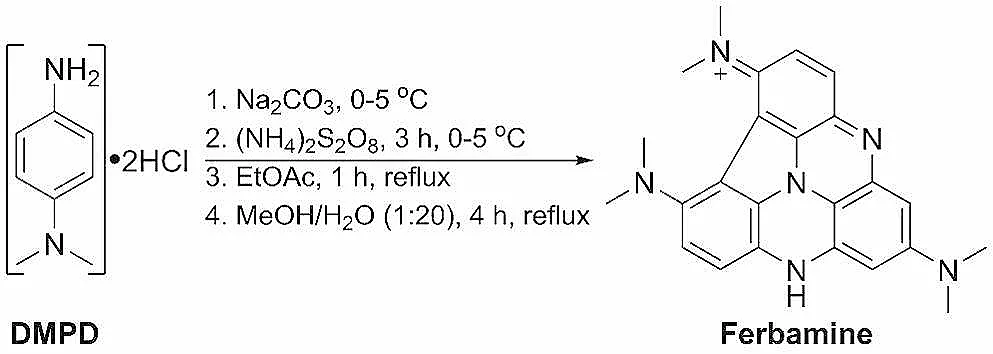
The overall scheme for Ferbamine synthesis

DMPD can be easily oxidised by laccase but lacks the stability required for long-term agar plate incubations. In this study, we pursued applying oxidation and nucleophilic addition reactions to oligomerise DMPD to a more suitable phenazine-type compound. Four constituents were prospected in the development and analysis of the substrate: (i) ease of synthesis; (ii) pH and thermal stability; (iii) sensitivity of the assay; (iv) toxicity to microorganisms. Overall, the aim was to develop a substrate which is easy to synthesise, stable over a wide pH range and at elevated temperatures and exhibits low toxicity to microorganisms. This was created by oligomerising DMPD to produce the Ferbamine substrate (Scheme 1).

Processes behind the oligomerization of phenylenediamines have yet to be completely understood and is an active field of research (Bassanini et al. 2023; Jiang et al. 2023). Therefore, in the scheme, we have presented one of the possible structures for Ferbamine based on the published works by Corbett (1999), Sousa et al. (2013, 2014), Modestov et al. (2004), and Jiang et al. (2020), and our analysis using the liquid chromatography combined with high-resolution mass spectrometry (Supplemental Fig. S4) followed by attenuated total reflectance Fourier transform infrared spectroscopy measurements (Supplemental Fig. S5). The initial Ferbamine synthesis step was derived from the synthesis of methylene blue (Berneth 2008), which entailed oxidation of the DMPD and an addition reaction with thiosulfate. In our case, thiosulfate was not used, allowing un-oxidized DMPD to react with its oxidized forms, resulting in oligomerization. After the initial oxidation step, refluxing was employed to create Ferbamine. Even though the synthesis procedure was easy to perform, the overall yield was about 0.27%; therefore, slight adjustments to the process were required to increase the yields.

For the initial synthesis step, oxidizing agent to DMPD ratio adjustment to 1:1 allowed to minimize the formation of by-products. Hydrogen peroxide proved to be unsuitable oxidizing agent because its use resulted in formation of additional by-products. The initial oligomerization of DMPD produced a precipitate containing a variety of oligomers and their isomers. Most of them were by-products; thus, refluxing of the precipitate with organic solvents was chosen for the study as the best method to remove the unwanted compounds. TLC analysis indicated that out of tested six solvents, EtOAc was the most effective at removing the by-products. Two types of silica gel chromatographies were also tested as a method for purification, but the basic nature of the by-products resulted in intense tailing of fractions. Chromatographic separation of these compounds may require specialized silica gel, retention modifiers or other types of mobile phase (Bidlingmeyer et al. 1982), which were not pursued in this work.

The second step of the overall synthesis process was performed by refluxing the obtained precipitate in dH_2_O. The optimal processing time was determined to be 4 h. The enabled pathway for Ferbamine synthesis was stable and easily repeatable; however, the overall conversion of DMPD to Ferbamine was still insufficient. It was assumed this might be related to the limited solubility of the precipitate in dH_2_O. Therefore, a strategy to increase the solubility was explored by using organic solvents entirely or their mixtures with dH_2_O. In this case, 5% MeOH showed the best performance (yield of 0.92%), while TLC analysis results indicated that Ferbamine did not form in mixtures with water content lower than 95%. A better understanding of the reaction mechanisms and elaborated optimization of the synthesis conditions could aid to achieve even higher yields.

The top priority in developing Ferbamine was to perpetrate the pH and thermal stability of the compound in screening assays. The UV-VIS spectra of native, partly oxidized or reduced Ferbamine solutions (Supplemental Fig. S6) demonstrated that Ferbamine exhibits high absorption at 550–570 nm range. Partially oxidized Ferbamine had weaker absorption at this range (~ 0.6 vs. ~ 0.3). Based on these observations, UV-VIS spectroscopy was employed to measure the stability of Ferbamine. According to the data in Fig. 2, Ferbamine was highly unstable at pH 8.0. The Ferbamine solution prepared in dH_2_O has not exposed a notable decrease in absorbance (> 86%) and remained highly stable at all temperatures. The stability of Ferbamine in the range of pH 3.5-6.0 in 50 mM Na-phosphate buffer was satisfactory; however, it was clear that buffer ions negatively affected the stability. In this work, the effect of other types of buffer ions on the stability of Ferbamine was not pursued. It is worth to mentioned that the distributors of the ABTS declare that it can be stored in a phosphate-citrate pH 5.0 buffer for two weeks at 4 °C and for one month at -20 °C (SIGMA-ALDRICH^®^ Technical bulletin). Another common substrate – syringaldazine – requires MeOH to increase its solubility making it incompatible with microorganisms (SIGMA-ALDRICH^®^ 2009). In essence, the results of our study have revealed that the stability of Ferbamine exceeds that of commercially available laccase substrates and this compound has potential as a substrate for methods requiring long-term incubations.

Ferbamine applicability for screening assays was carried out using Ferbamine-agar plates. Substrate-agar plate methods are very convenient for ascertaining functionality and do not require spectroscopy equipment such as a spectrophotometer or well plate reader. Initial test with the commercially available laccase Novozymes^®^ 51,003 displayed pale yellow area with a purple halo. The absorption maximum of the oxidised Ferbamine is in the near UV-400 nm range (Supplemental Fig. S6), which can be seen as a pale yellow colour in the central area. The colour of the halo was slightly different depending on the amount of enzyme added. Overall, it indicated that it is possible to distinguish that oxidation has occurred visually.

Further, the screening with Ferbamine as a substrate was applied to survey laccase activity in extracts from terrestrial fungi. For most extracts, the correlation between the results of the Metol and Ferbamine assays concurred; however, five extracts showed substantial deviation. Extracts N°6, N°33 and N°50 had high laccase activity in the Metol assay (1.13–5.25 U ml^-1^) but were only slightly or not detectable on the Ferbamine plates. N°16 and N°24 displayed noticeable activities on Ferbamine plates but did not expose any activity in the Metol assay. These deviations may have emerged due to the enzymes’ specificity, e.g., laccase’s selectivity to substrates or a possible dependence on pH or other factors. A similar inconsistency has been documented by Erden et al. (2009) for the laccase activity screening in fungi with guaiacol, ABTS and syringaldazine (N°40). Out of 40 species presented in Table 2, laccase activity has been reported for 14 species in the literature. In this survey, eight species exposed the laccase activity. Still, in other studies, these species were reported as laccase-negative ones. 18 species, to our knowledge, have not been tested before and displayed laccase-like activity using Ferbamine and/or metol. In summary, Ferbamine displayed persuasive performance in detecting laccase activity in extracts from environmental samples and proved to be a suitable substrate for screening activities on agar plates.

The foreseen intention is also to apply Ferbamine in protein engineering for functional analysis. Therefore, the tests on the toxicity of Ferbamine to microorganisms were performed on strains which are widely used as hosts for gene engineering – gene expression and the creation of genomic or metagenomic libraries. The data on *E. coli*, *P. putida* and five additionally tested Gram-negative bacteria (*A. denitrificans*, *D. acidovorans*, *P. aeruginosa*, *A. faecalis*, *A. calcoaceticus*) suggested that Gram-negative bacteria were more resistant to Ferbamine. This observation could be explained by the fact that Gram-negative bacteria are more resistant to chemical agents because their outer membrane acts as an additional barrier to molecule entry (Jones 2017; Zhang 2022).

To sum up, despite some limitations, Ferbamine is a suitable substrate for experiments involving microorganisms and can be applied in high-throughput screening of laccase activity on agar plates. Further modifications of Ferbamine could yield to a substrate with even greater sensitivity to laccase activity or better biocompatibility. Many enzymes with high potential lack quality substrates with such characteristics as high thermal and pH stability, considerable sensitivity and selectivity, acceptable solubility in water, and vast speed of catalysis, and laccase is not an exception. Through this work, we have presented the development and investigation of a phenazine substrate Ferbamine.

## Electronic supplementary material

Below is the link to the electronic supplementary material.

Supplementary Material 1

## Data Availability

The authors declare that the data supporting the findings of this study are available within the paper and its Supplementary Information files. Should any raw data files be needed in another format they are available from the corresponding author upon reasonable request.
