# Supplementary material for: Synthesis and application of a phenazine class substrate for high-throughput screening of laccase activity
Source: Appl Microbiol Biotechnol. 2024 Jan 9;108(1):66. doi: 10.1007/s00253-023-12958-7 (PMC10776486; doi:10.1007/s00253-023-12958-7)
Supplement: Supplementary file 1 — Supplementary Material 1 [file 253_2023_12958_MOESM1_ESM.pdf]

Applied Microbiology and Biotechnology

SUPPORTING INFORMATION

Synthesis and application of a phenazine class substrate for high-throughput screening of laccase activity

Justinas Babinskas[1], Jerica Sabotič[2], and Inga Matijošytė[1]*

[1] Vilnius University, Life Sciences Center, Institute of Biotechnology, Sector of Applied Biocatalysis, Saulėtekio ave. 7, LT-10257, Vilnius, Lithuania.
[2] Department of Biotechnology, Jožef Stefan Institute, Jamova cesta 39, 1000 Ljubljana, Slovenia.
* Corresponding author: Inga Matijošytė; email inga.matijosyte@bti.vu.lt

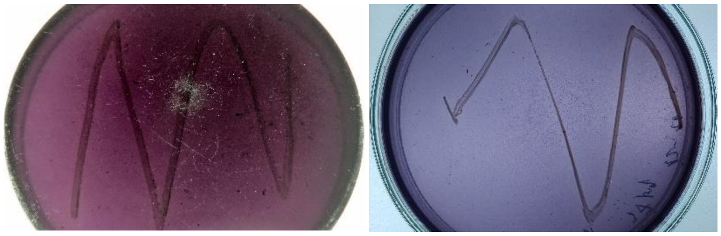

Figure S1. Results of *E. coli* and *P. putida* growth on 37.5 µg ml^{-1} Ferbamine-LB agar and Ferbamine-TS agar plates after 10 h of incubation, respectively.

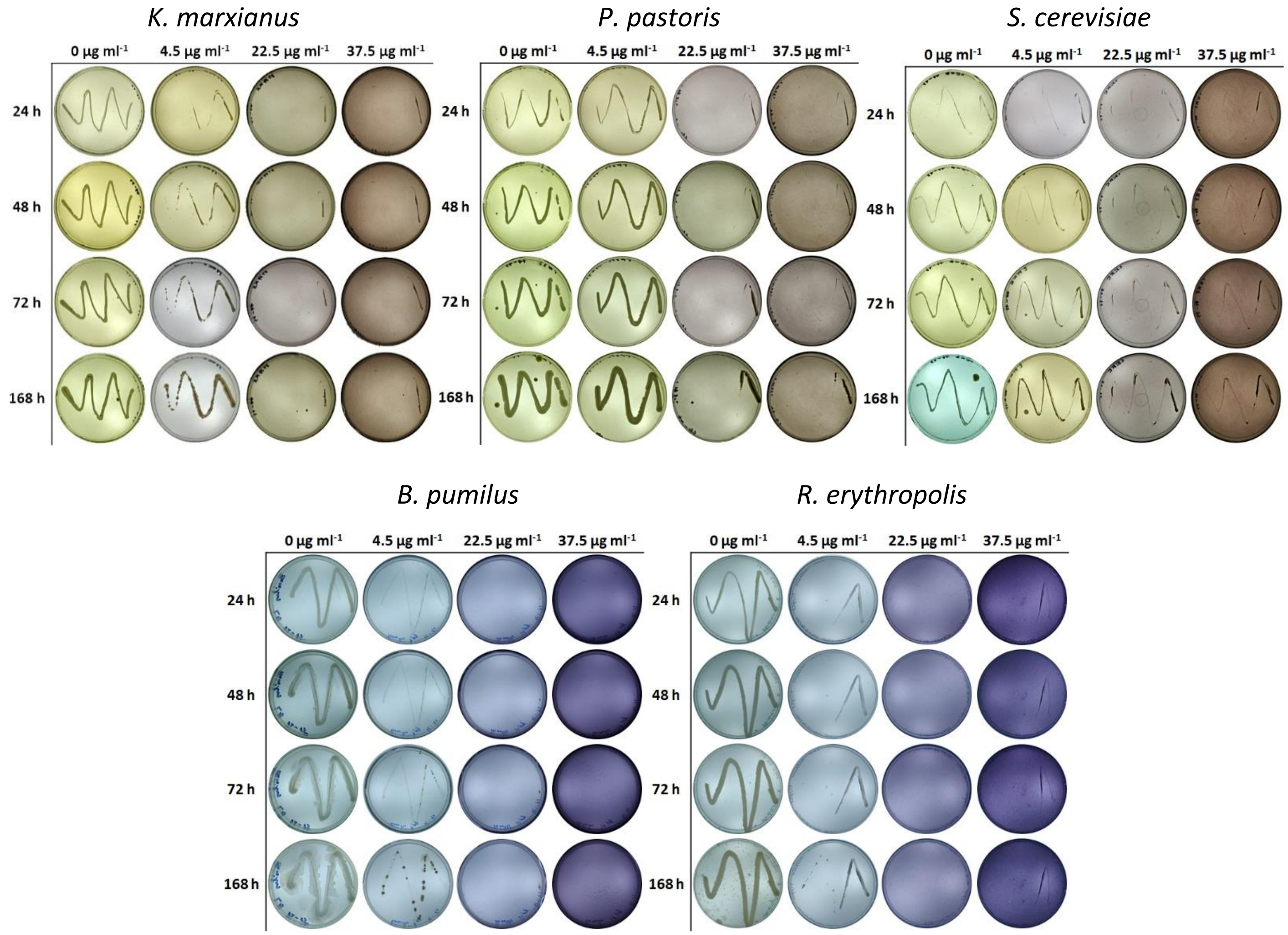

Figure S2. Results of Ferbamine concentration (0-37.5 µg ml^{-1}) influence on the growth of *K. lactis, P. pastoris, S. cerevisiae, B. pumilus* and *R. erythropolis* growth on 0-37.5 µg ml^{-1} Ferbamine and YEPG, LB or nutrient agar plates.

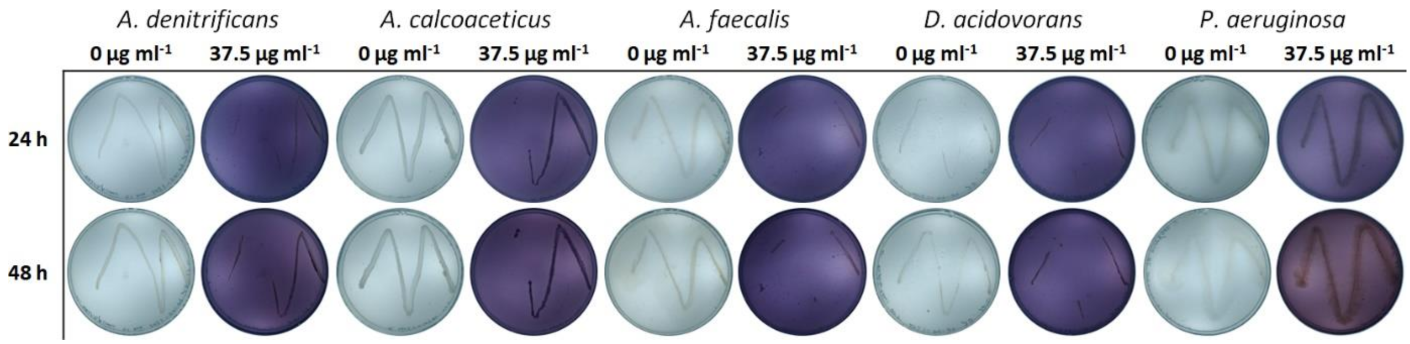

Figure S3. Results of Ferbamine concentration (0-37.5 µg ml^{-1}) influence on the growth of *R. erythropolis, A. denitrificans, D. acidovorans, P. aeruginosa, A. faecalis* and *A. calcoaceticus* growth 37.5 µg ml^{-1} Ferbamine-nutrient agar and control plates.

Liquid chromatography (LC) high-resolution mass spectrometry (HRMS) analysis of Ferbamine synthesis product. The chromatography and mass spectrometry were carried out at Institute of Chemistry, Vilnius University (Lithuania), using Dual-Esi Q-TOF 6250 spectrometer (Agilent Technologies, Santa Clara, USA). Data analysis was performed by the authors' group.

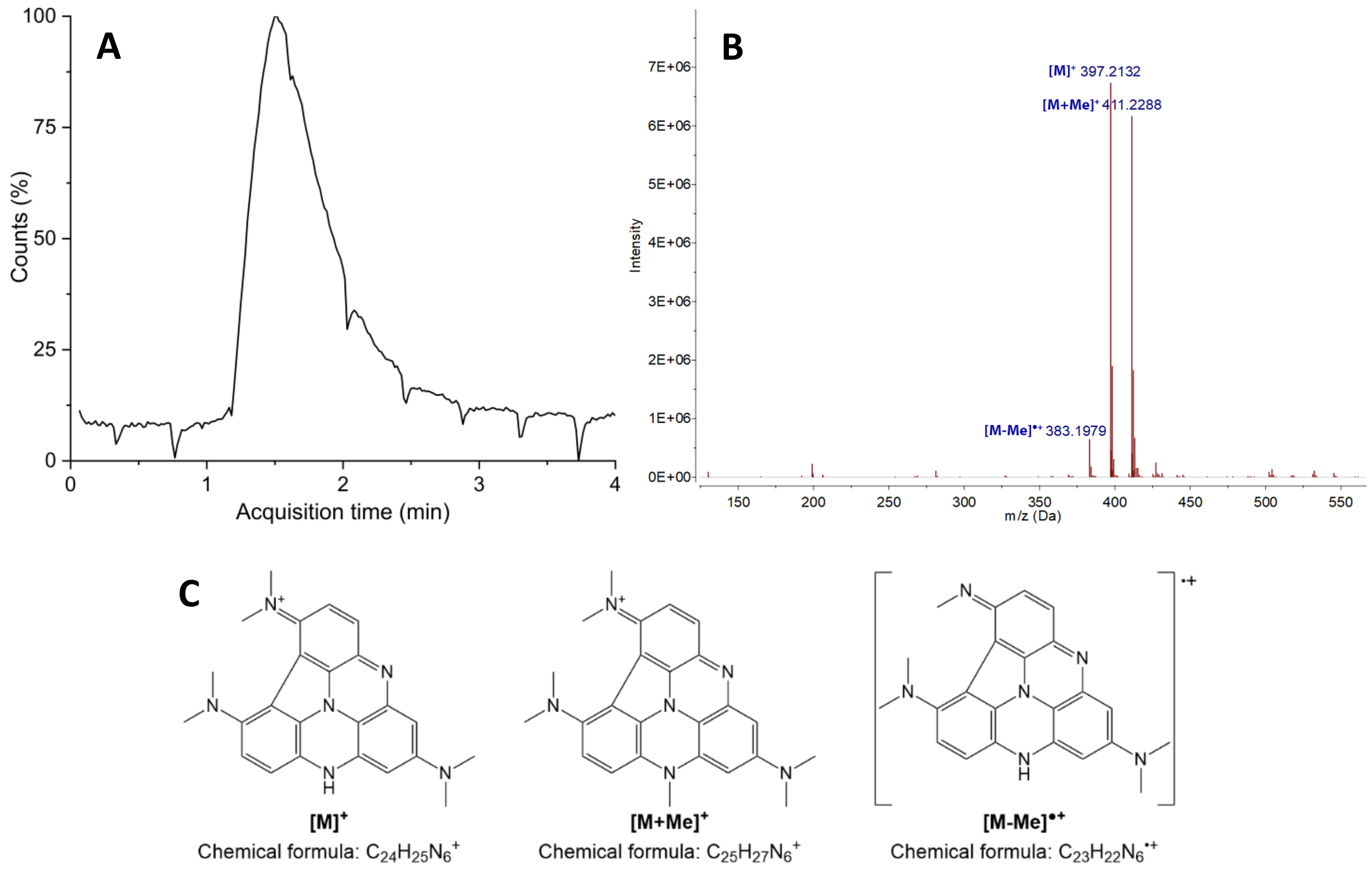

Figure S4. Results of Ferbamine LC-HRMS analysis. **A** – LC chromatogram; **B** – HRMS spectra at chromatography acquisition (retention) time 1.515 min; **C** – predicted chemical formula and assigned molecular structures to HRMS signals.

Attenuated total reflectance Fourier-transform infrared spectroscopy (ATR-FTIR) analysis of Ferbamine synthesis product.

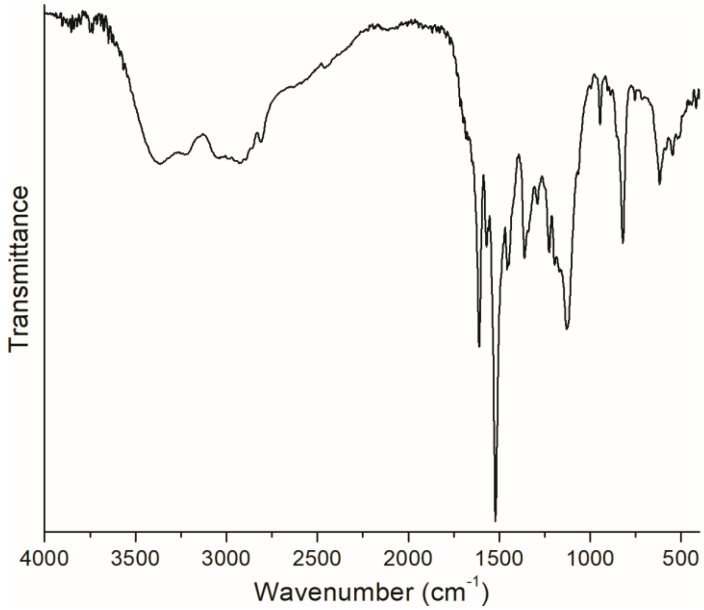

Figure S5. ATR-FTIR spectra of Ferbamine.

Ultraviolet-Visible (UV-VIS) light spectroscopy of native, partially reduced or oxidized Ferbamine solutions.

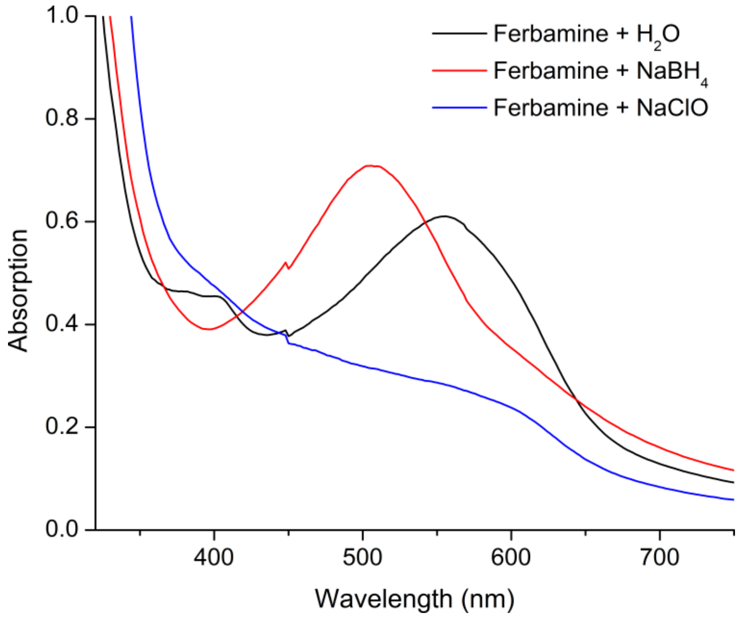

Figure S6. UV-VIS spectra of partially reduced (Ferbamine + $NaBH_4$), oxidized (Ferbamine + NaClO) and control (Ferbamine + H_2O) Ferbamine solutions.

Table S1. Result of laccase activity screening in the collection of extracts from terrestrial fungi.

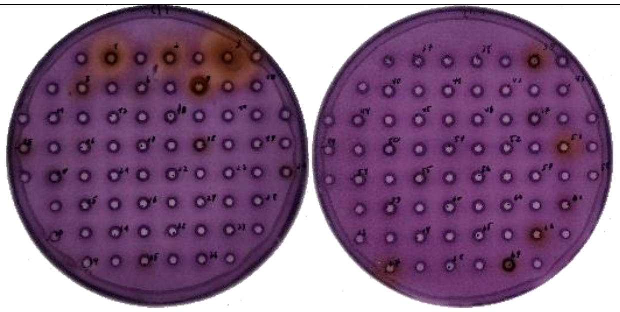

Extract No.	Species	Assays<br>Metol [U ml^{-1}][a]	Ferbamine	Previously reported laccase[b]
3	*Clitocybe nebularis*	9.00	+++	Yes (Asav 2021)
39	*Psilocybe cubensis*	5.40	+++	Yes (Lenz et al. 2020)
6	*Cortinarius violaceus*	5.25	+	No (Chen et al. 2003)
2	*Macrolepiota procera*	4.80	+++	Yes (Luis et al. 2004)
50	*Gomphidius glutinosus*	3.90	-	Not tested
53	*Pleurotus eryngii*	2.70	+++	Yes (Guo et al. 2017)
1	*C. nebularis*	2.55	+++	Yes (Asav 2021)
67	*Rhodocybe gemina*	1.65	+	Not tested
15	*Tricholoma tigrinum*	1.65	+	Not tested
66	*Tricholoma saponaceum*	1.50	++	Yes (Khaund and Joshi 2014)
33	*Lentinula edodes*	1.13	-	Yes (Yano et al. 2009)
5	*Ganoderma lucidum*	1.05	++	Yes (Jeon et al. 2008)
55	*Gymnopilus penetrans*	0.90	+	No (Marr et al. 1986)
9	*Cortinarius caperatus* (*Rozites caperata*)	0.83	+++	Not tested
62	*Amanita vaginata*	0.83	+	Not tested
47	*Anthurus archeri* (*Clathrus archeri*)	0.75	+	Not tested
18	*Pleurotus ostreatus* (summer strain)	0.75	+	Yes (Park and Park 2014)
35	*Clitocybe geotropa*	0.68	+	Not tested
54	*Amanita spissa*	0.68	-	No (Chen et al. 2003)
70	*A. vaginata*	0.68	-	Not tested
51	*Tricholomopsis rutilans*	0.68	-	Not tested
59	*Clavariadelphus pistillaris*	0.60	+	Yes (Marr et al. 1986)
40	*Ramaria stricta*	0.53	-	Yes (ABTS, guaiacol), No (syringaldazine) (Erden et al. 2009)
20	*P. ostreatus* (winter strain)	0.45	+	Yes(Park and Park 2014)
36	*Russula alutacea*	0.45	+	Not tested
57	*Clitocybe geotropa*	0.38	-	Not tested
31	*Paxilllus atrotomentosus*	0.38	-	Not tested
69	*Cortinarius multiformis*	0.30	++	Not tested
32	*Clitocybe gibba*	0.30	-	Not tested

28	*Lactarius necator*	0.23	-	Not tested
63	*Amanita rubescens*	0.15	-	No (Hutchison 1990)
58	*Craterellus cornucopioides*	0.15	-	Not tested
43	*P. ostreatus*	0.15	-	Yes (Park and Park 2014)
42	*Xerocomus badius*	0.15	-	No (Hutchison 1990)
65	*Agaricus bisporus*	0.08	-	Yes (Mayolo-Deloisa et al. 2011)
41	*A. rubescens*	0.08	-	No (Hutchison 1990)
42	*A. rubescens*	0.08	-	No (Hutchison 1990)
46	*P. atrotomentosus*	0.08	-	Not tested
24	*Amanita excelsa*	-	+	Not tested
16	*Macrolepiota rachodes*	-	+	No (Luis et al. 2004)
8	Amanita citrina	-	-	No (Hutchison 1990)
61	*A. citrina*	-	-	No (Hutchison 1990)
64	*A. citrina*	-	-	No (Hutchison 1990)
37	*A. rubescens*	-	-	No (Hutchison 1990)
60	*A. spissa*	-	-	No (Chen et al. 2003)
68	*A. spissa*	-	-	No (Chen et al. 2003)
21	*Armillaria borealis*	-	-	Not tested
22	*Boletus badius*	-	-	Not tested
11	*Boletus erythropus*	-	-	Not tested
25	*Coprinus comatus*	-	-	Yes (Bao et al. 2013)
34	*Fomitopsis pinicola*	-	-	Yes (Park and Park 2014)
23	*Kuehneromyces mutabilis*	-	-	Yes (Gramss 2020)
14	*L. necator*	-	-	Not tested
19	*L. edodes*	-	-	Yes (Yano et al. 2009)
56	*Lycoperdon perlatum*	-	-	Yes(Harkin et al. 1974)
27	*Sparassis crispa*	-	-	Not tested
10	*Suillus bovinus*	-	-	No(Timonen and Sen 1998)
38	*Suillus granulatus*	-	-	Yes (Cullings et al. 2008)
17	*Suillus luteus*	-	-	No (Mucha 2011)
30	*Suillus variegatus*	-	-	Not tested
26	*Tricholoma sulphureum*	-	-	Not tested

[a] 1 U is defined as 1 μmol of Metol catalysed per 1 min, at pH 5.0 and 20 °C.

[b] Yes – laccase gene or activity has been previously reported in the species; No – species has been tested, but laccase genes or activity was not detected; Not tested – species has not been tested for laccase genes or activity.

References

Asav E (2021) Sensitive determination of 3,4-dihydroxyl-L-phenylalanine by a cloud funnel mushroom (*Clitocybe nebularis* (Batsch), P. Kumm.) homogenate-based amperometric biosensor. Trak Univ J Nat Sci 22:255–262. https://doi.org/10.23902/trkjnat.969982

Bao S, Teng Z, Ding S (2013) Heterologous expression and characterization of a novel laccase isoenzyme with dyes decolorization potential from *Coprinus comatus*. Mol Biol Rep 40:1927–1936. https://doi.org/10.1007/s11033-012-2249-9

Chen DM, Bastias BA, Taylor AFS, Cairney JWG (2003) Identification of laccase-like genes in ectomycorrhizal basidiomycetes and transcriptional regulation by nitrogen in *Piloderma byssinum*. New Phytol 157:547–554. https://doi.org/10.1046/j.1469-8137.2003.00687.x

Cullings K, Ishkhanova G, Henson J (2008) Defoliation effects on enzyme activities of the ectomycorrhizal fungus *Suillus granulatus* in a *Pinus contorta* (lodgepole pine) stand in Yellowstone National Park. Oecologia 158:77–83. https://doi.org/10.1007/s00442-008-1119-6

Erden E, Ucar MC, Gezer T, Pazarlioglu NK (2009) Screening for ligninolytic enzymes from autochthonous fungi and applications for decolorization of remazole marine blue. Braz J Microbiol 40:346–353. https://doi.org/10.1590/S1517-83822009000200026

Gramss G (2020) Aspects determining the dominance of *Fomitopsis pinicola* in the colonization of deadwood and the role of the pathogenicity factor oxalate. Forests 11:290. https://doi.org/10.3390/f11030290

Guo C, Zhao L, Wang F, Lu J, Ding Z, Shi G (2017) *β*-Carotene from yeasts enhances laccase production of *Pleurotus eryngii* var. *ferulae* in co-culture. Front Microbiol 8:1101. https://doi.org/10.3389/fmicb.2017.01101

Harkin JM, Larsen MJ, Obst JR (1974) Use of syringaldazine for detection of laccase in sporophores of wood rotting fungi. Mycologia 66:469–476. https://doi.org/10.2307/3758490

Hutchison LJ (1990) Studies on the systematics of ectomycorrhizal fungi in axenic culture. III. Patterns of polyphenol oxidase activity. Mycologia 82:424–435. https://doi.org/10.2307/3760013

Jeon J-R, Murugesan K, Kim Y-M, Kim E-J, Chang Y-S (2008) Synergistic effect of laccase mediators on pentachlorophenol removal by *Ganoderma lucidum* laccase. Appl Microbiol Biotechnol 81:783–790. https://doi.org/10.1007/s00253-008-1753-2

Khaund P, Joshi SR (2014) Enzymatic profiling of wild edible mushrooms consumed by the ethnic tribes of India. J Korean Soc Appl Biol Chem 57:263–271. https://doi.org/10.1007/s13765-013-4225-z

Lenz C, Wick J, Braga D, García-Altares M, Lackner G, Hertweck C, Gressler M, Hoffmeister D (2020) Injury-triggered blueing reactions of *Psilocybe* “magic” mushrooms. Angew. Chem Int Ed 59:1450–1454. https://doi.org/10.1002/anie.201910175

Luis P, Walther G, Kellner H, Martin F, Buscot F (2004) Diversity of laccase genes from basidiomycetes in a forest soil. Soil Biol Biochem 36:1025–1036. https://doi.org/10.1016/j.soilbio.2004.02.017

Marr CD, Grund DW, Harrison KA (1986) The taxonomic potential of laccase and tyrosinase spot tests. Mycologia 78:169–184. https://doi.org/10.2307/3793162

Mayolo-Deloisa K, Machín-Ramírez C, Rito-Palomares M, Trejo-Hernández MR (2011) Oxidation of polycyclic aromatic hydrocarbons using partially purified laccase from residual compost of *Agaricus bisporus*. Chem Eng Technol 34:1368–1372. https://doi.org/10.1002/ceat.201000205

Mucha J (2011) Changes in hyphal morphology and activity of phenoloxidases during interactions between selected ectomycorrhizal fungi and two species of *Trichoderma*. Antonie van Leeuwenhoek 100:155–160. https://doi.org/10.1007/s10482-011-9556-3

Park N, Park S-S (2014) Purification and characterization of a novel laccase from *Fomitopsis pinicola* mycelia. Int J Biol Macromol 70:583–589. https://doi.org/10.1016/j.ijbiomac.2014.06.019

Timonen S, Sen R (1998) Heterogeneity of fungal and plant enzyme expression in intact Scots pine–*Suillus bovinus* and –*Paxillus involutus* mycorrhizospheres developed in natural forest humus. New Phytol 138:355–366. https://doi.org/10.1046/j.1469-8137.1998.00103.x

Yano A, Kikuchi S, Nakagawa Y, Sakamoto Y, Sato T (2009) Secretory expression of the non-secretory-type *Lentinula edodes* laccase by *Aspergillus oryzae*. Microbiol Res 164:642–649. https://doi.org/10.1016/j.micres.2008.12.001
